# Bacteriological profile and antimicrobial susceptibility patterns of blood culture isolates among febrile patients in Mekelle Hospital, Northern Ethiopia

**DOI:** 10.1186/s40064-015-1056-x

**Published:** 2015-07-03

**Authors:** Araya Gebreyesus Wasihun, Letemichael Negash Wlekidan, Senay Aregawi Gebremariam, Tsehaye Asmelash Dejene, Abadi Luel Welderufael, Tadesse Dejenie Haile, Saravanan Muthupandian

**Affiliations:** Department of Medical Microbiology and Immunology, Institute of Biomedical Sciences, College of Health Sciences, Mekelle University, 1871 Mekelle, Ethiopia; Department of Internal Medicine, School of Medicine, College of Health Sciences, Mekelle University, Mekelle, Ethiopia; Department of Pediatric Medicine, School of Medicine, College of Health Sciences, Mekelle University, Mekelle, Ethiopia; Department of Biology, College Natural and Computational Sciences, Mekelle University, Mekelle, Ethiopia

**Keywords:** Prevalence, Blood culture, Bacterial isolates, Febrile patients, Antimicrobial susceptibility pattern, Multidrug resistance, Mekelle hospital

## Abstract

Bacterial bloodstream infections are a major public health problem, which leads to high morbidity and mortality of patients. On time diagnosis and appropriate medication will be the best way to save the lives of affected ones. The aim of the present study was to determine the bacterial profile of bloodstream infections and their antibiotic susceptibility pattern in Mekelle Hospital. Cross sectional study method was carried out in 514 (269 females and 245 males) febrile patients in Mekelle hospital from March to October 2014. Standard bacteriological methods were used for blood collection, bacterial isolation and antimicrobial susceptibility pattern. Out of the total 514 febrile patients, 144 (28%) culture positive were isolated. *Staphylococcus aureus* 54 (37.5%), Coagulase-negative staphylococci 44 (30.6%), *Escherichia coli* 16 (3.1%), *Citrobacter* spp. 9 (1.7%) and *Salmonella typhi* 8 (1.6%) were the most dominant isolates, collectively accounting for >90% of the isolates. Antimicrobial resistance pattern for gram positive and gram negative bacteria was 0–83.3% and 0–100%, respectively. High resistance was seen to Trimethoprim-sulphamethoxazole 101 (70.1%), Oxacillin 65 (62.5%), Ceftriaxone 79 (58.9%) and Doxycycline 71 (49.3%). Fifty-nine percent of the isolated bacteria in this study were multi drug resistant. Most bacterial isolates were sensitive to Gentamicin, Ciprofloxacin and Amoxicillin clavulanic acid. All gram positive isolates in this current study were sensitive to vancomycin. Prevalence of bacterial isolates in blood was high. It also reveals isolated bacteria species developed multi drug resistance to most of the antibiotics tested, which highlights for periodic surveillance of etiologic agent, antibiotic susceptibility to prevent further emergence and spread of resistant bacterial pathogens.

## Background

Bloodstream infection (BSI) remains one of the most important causes of morbidity and mortality globally (Zenebe et al. 2011). Though the problem is still common in developed nations (Diekma et al. 2002), the burden is high in sub Saharan countries with 53% children mortality rate (Aiker et al. 2011).

Many bacteria have been reported which cause bacteraemia with variation in distribution from place to place (Daniel et al. 2006; Asrat and Amanuel 2001; James et al. 2004; Gohel et al. 2014; Rina et al. 2007). Infection by these organisms causes prolonged patient hospitalization, increased healthcare costs and mortality rate of patients (Tziamabos and Kasper 2005).

Treatment of bacteraemia is usually done by timely administration of appropriate antibiotics. However, since many bacterial pathogens have developed resistance to most of the antibiotics, it has become a serious health problem with many economic and social inferences all over the world (JoAnn 2009). Researches in Ethiopia revealed that there is high bacterial drug resistance to commonly used antibiotics mainly due to the lack of national guideline for antibiotic use, absence of good laboratory facilities to do antimicrobial drug susceptibility test. As a result clinicians use empirical way to treat their patients, there is also high self treatment of humans, and animals without prescription of doctors. These all would lead to emergence and rapid dissemination of resistance (Zenebe et al. 2011).

Research findings have reported that inappropriate treatment of BSI aggravates to increased mortality of patients and emerging of drug resistance strains (JoAnn 2009; Zenebe et al. 2011; Ali and Kebede 2008). In Ethiopia there are only a few studies on organisms involved in bloodstream bacterial infection and their susceptibility pattern (Dagnew et al. 2013; Asrat and Amanuel 2001; Zenebe et al. 2011; Ali and Kebede 2008); however, we could not get any published data in the study area and in the region as a whole. Therefore, we conducted this study to determine the common bacterial agents associated with bacteraemia and their antimicrobial susceptibility patterns in febrile patients in Mekelle hospital in Tigray, Northern Ethiopia.

## Methods

### Study design, study area and sampling process

Prospective cross-sectional study design was used to conduct the study among febrile out patients from March to October 2014. Mekelle Hospital, which is located 783 km North of Addis Ababa, is the largest hospital in the region giving services since the last 58 years. Sample size was calculated considering 24.2% prevalence (Ali and Kebede 2008), 5% precision, and 95% confidence level; with 15% contingency, thus a total of 514 febrile patients were included. Patients who were on antibiotic within the last 2 weeks of visiting the hospital were excluded from this study.

### Data collection and laboratory procedures

About 5 ml of venous blood for adults and 2–3 ml for children was collected aseptically using 70% alcohol and 2% tincture of iodine and transferred into a bottle containing 45 ml of tryptic soy broth sterile culture medium (BBL™ USA). Blood culture broths were then transported within 30 min to Ayder referral hospital microbiology laboratory and incubated at 37°C.

### Bacterial identification

Blood culture broths were checked for sign of bacterial growth (turbidity, haemolysis, clot formation) daily up to 7 days. Bottles which showed signs of growth were further processed by gram stain and sub-culture was made onto blood agar, MacConkey agar, Manitol salt agar (all Oxoid Ltd, UK) and incubated at 37°C for 24 h. Blood culture broths with no bacterial growth after 7 days were sub-cultured before being reported as a negative result. Bacterial isolates were identified by colony morphology, gram staining reaction, biochemical tests using Catalase test, Coagulase test, Triple Sugar Iron agar (TSI) (OXOID, UK),citrate utilization test (BBL™),Urease test (BBL™) and Motility Indole Lysine (MIL) (BBL™) test using the standard procedure for bacterial identification.

### Antimicrobial susceptibility test

Following identification of the bacterial isolates, disc diffusion method was done according to Clinical Laboratory Standards Institute (CLSI) guidelines (M100 S24, 2014). The antibiotics discs and their concentrations were: Amoxicillin-clavunilic acid; (30 µg) (OXOID, UK), Ceftriaxone, (30 µg) (BBL™),Vancomycin (30 µg) (BBL™), Oxacillin (1 µg) (BBL™^)^, Ciprofloxacin (5 µg) (BBL™), Gentamicin (120 µg) (BBL™), Norfloxacin (10 µg) (OXOID), Doxycycline (30 µg) (OXOID UK), Erythromycin (15 µg) (BBL™), Nitrofurantonin (300 µg) (BBL™) and Trimethoprim-sulphamethoxazole (25 µg) (BBL™ USA). We selected these antimicrobial agents as they are available and frequently prescribed for the management of bacterial infections in the hospital and in Ethiopia as whole.

*S. aureus* and CoNS isolates which were found to be vancomycin resistant by disk diffusion methods were sub-cultured in skim milk agar and stored at −60°C for further Vancomycin minimum inhibitory concentration (MIC) determination by broth micro dilutions (BMD) methods (CLSI, M100 S24, 2014).

### Quality control

Reference strains *E*. *coli* (ATCC 25922) and *S. Aureus* (ATCC 25923) were used as a control reference strains for identifications and drug susceptibility testing. Negative control was done by randomly taking the prepared culture media and incubating over nigh to see for any growth. In this study multi-drug resistance was defined as simultaneous resistance to more than two antimicrobial agents (Magiorakos et al. 2012).

### Data analysis

SPSS version 20 software was used for statistical analysis. Chi square test (χ^2^) was used to determine relationship between dependent and independent variable. P value <0.05 was used to indicate significant association.

## Results

From the total 514 febrile patients, 269 (52.3%) were females and 245 (47.7%) males. Their age ranges from 1–80 years [mean 28.912 ± 1.46 (SD)]. Two hundred seventy-two (52.9%) of the participants were in the age range of 15–29 years. A total of 144 (28%) bacterial strains were isolated. Predominant isolates were *S. aureus* 54 (10.3%), Coagulase negative staphylococci (CoNS) 44 (8.5%), *E. coli* 16 (3.1%), *Citrobacter* spp. 9 (1.7%) and *S. typhi* 8 (1.6%) (Table 1).

**Table 1:** Frequencies of bacterial species isolated from blood cultures of febrile patients attending Mekelle hospital, Northern Ethiopia (March–October 2014)

Bacterial species	No.	% of the total patients (n = 514)
*S. aureus*	54	10.3
CoNS	44	8.5
*E. coli*	16	3.1
*Citrobacter* spp.	9	1.7
*S. typhi*	8	1.6
*S. pyogenes*	5	1.0
*Acinetobacter* spp.	4	0.8
*S. paratyphi*	2	0.4
*P. mirabilis*	1	0.2
*Klebsiella* spp.	1	0.2
*P. aeruginosa*	1	0.2
Total	144	28

*CoNS* coagulase negative staphylococcus.

More bacteria were isolated form females than males, though it was not statistically significant (P = 0.06, χ^2^ = 3.47, df = 1). The spectrum of bacteria varies with the age of patients, where 48.6% of the bacteria isolates were found in the age group of 15–29 years but not statistically significant associated (P = 0.54, χ^2^ = 46.4, df = 48).

In vitro antibiotic susceptibility of the bacterial isolates (Table 2) showed resistance for gram positive bacteria from 0 to 83%. Thirty-six (66.7%) of *S. aureus* isolates were resistant to Trimethoprim-sulphamethoxazole, 31 (57.4%) to Ceftriaxone, 29 (53.7%) to Doxycycline and 38 (70.4%) to Oxacillin. Thirty-six (81.8%) and 27 (61.4%) resistance was seen by CoNS to Trimethoprim-sulphamethoxazole and Doxycycline, respectively. All *S.aureus* and CoNS isolates were sensitive to vancomycin. Over all, high resistance was seen by gram positive isolates to Trimethoprim-sulphamethoxazole (74%) and Doxycycline (57.7%). Amoxicillin clavulanic acid was an effective antibiotic for gram positive bacteria next to Vancomycin in our study.

**Table 2:** Antimicrobial susceptibility pattern of bacterial isolates from blood culture of febrile patients at Mekelle hospital, Northern Ethiopia (March–October 2014) no. (%)

Organism	Bacterial isolates resistant to each drug tested (%)									
	AMC	CRO	CN	DO	CIP	SXT	E	OX	NOR	F
*S. aureus*	7 (13)	31 (57.4)	18 (33.3)	29 (53.7)	21 (38.9)	36 (66.7)	24 (44.4)	38 (70.4)	18 (33.3)	NA
CoNS	5 (11.4)	22 (50)	8 (18.1)	27 (61.4)	11 (25)	36 (81.8)	18 (40.9)	27 (61.4)	13 (29.5)	NA
*S. pyogenes*	1 (16.7)	1 (16.7)	0	4 (66.7)	1 (16.7)	5 (83.3)	1 (16.7)	0	1 (16.7)	NA
*E. coli*	1 (6.7)	9 (60)	2 (13.3)	6 (40)	1 (6.7)	10 (6.7)	NA	NA	9 (60)	4 (26.7)
*Citrobacter* spp.	2 (25)	4 (50)	2 (25)	4 (50)	1 (12.5)	3 (37.5)	NA	NA	6 (75)	1 (12.5)
*Acinetobacter* spp.	1 (20)	4 (80)	0	2 (40)	0	3 (60)	NA	NA	2 (40)	0
*S. typhi*	0	3 (37.5)	2 (25)	4 (50)	1 (12.5)	4 (50)	NA	NA	6 (75)	2 (25)
*S. paratyphi*	0	1 (50)	1 (50)	1 (50)	1 (50)	1 (50)	NA	NA	1	1 (50)
*Klebsiella* spp.	0	0	0	0	0	0	NA	NA	1 (100)	1 (100)
*P. mirabilis*	0	0	0	0	0	0	NA	NA	0	1 (100)
*P. aeruginosa*	0	0	1 (100)	1 (100)	0	0	NA	NA	0	0
Total	17 (11.8)	75 (52)	34 (23.6)	78 (54.2)	37 (25.7)	89 (61.8)	45 (31.3)	65 (62.5)	56 (38.9)	10 (22.2)

*AMC* amoxicillin clavulanic acid, *CRO* ceftriaxone, *CN* gentamicin, *DO* doxycycline, *CIP* ciprofloxacin, *SXT* trimethoprim-sulphamethoxazole, *E* erythromycin, *OX* oxacillin, *NOR* norfloxacin, *F* Nitrofurantonin, *NA* not applicable.

Antimicrobial resistance level of gram negative bacteria in this study was from 0 to 100%. *E. coli* was resistant to Ceftriaxone and Nitrofurantonin (60% each), *Acinetobacter* spp. were 80% resistant to Ceftriaxone and 60% to Trimethoprim-sulphamethoxazole. *S. typhi* isolates were resistant to Doxycycline, Trimethoprim-sulphamethoxazole and Nitrofurantonin (75% each). Over all, high resistance was seen by gram negative isolates to Nitrofurantonin 53.3% and Ceftriaxone 46.7%. Amoxicillin clavulanic acid and Ciprofloxacin were, however, effective for gram negative bacteria.

Antibiogram pattern of the isolates in this study showed that, *S. aureus* 34 (63%), CoNS 27 (61.2%), *S. pyogenes* 3 (50%), *Citrobacter* spp. 5 (62.5%), *E. coli* 8 (53.3%) and *Acinetobacter* spp. 4 (80%) showed multiple drug resistance (MDR). In general, 59% of the isolates in our study developed multidrug resistance to different antibiotics (Table 3).

**Table 3:** Multiple drug resistance patterns of gram positive and gram negative bacteria in blood of febrile patients attending Mekelle hospital, Northern Ethiopia, (March–October 2014)

Organisms	Antibiogram pattern no. (%)								
	R0	R1	R2	R3	R4	R5	R6	R7	R8
*S. aureus* (n = 54)	4 (7.4)	10 (18.5)	5 (9.3)	6 (11.1)	6 (11.1)	5 (9.3)	8 (14.8)	5 (9.3)	4 (7.4)
CoNS (n = 44)	4 (9.1)	4 (9.1)	9 (20.5)	9 (20.5)	6 (13.6)	3 (6.8)	4 (9.1)	4 (9.1)	1 (2.3)
*S. pyogenes* (n = 6)	–	1 (16.7)	2 (33.3)	1 (16.7)	1 (16.7)	1 (16.7)	–	–	–
*E. coli* (n = 15*)*	–	5 (.3)	2 (13.3)	3 (20)	4 (26.7)	–	1 (6.7)	–	–
Citrobacter spp. (n = 8)	–	2 (.25)	1 (12.5)	2 (.25)	3 (37.5)	–	–	–	–
*S. typhi* (n = 8)	–	2 (.25)	1 (12.5)	2 (.25)	1 (12.5)	–	1 (12.5)	–	–
*Acinetobacter* spp. (n = 5)	1 (20)	–	2 (40)	–	2 (20)	–	–	–	–
*S. paratyphi* (n = 2)	–	–	1 (50)	–	–	–	1 (50)	–	–
*P. mirabilis* (n = 1)	–	1 (100)	–	–	–	–	–	–	–
*Klebssiels* spp. (n = 1)	–	–	1 (100)	–	–	–	–	–	–
*P. aeruginosa* (n = 1)	–	–	–	1 (100)	–	–	–	–	–
Total (144)	9 (6.25)	25 (17.4)	24 (16.7)	24 (16.7)	23 (16)	9 (6.25)	15 (10.4)	9 (6.25)	5 (3.5)

*CoNS* coagulase negative *staphylococci*, *R0* sensitive to all antibiotics tested; *R1*, *R2*, *R3*, *R4*, *R5*, *R6*, *R7*, *R8*, *R9* resistant to one, two, three, four, five, six, seven, eight and nine antibiotics, respectively.

MIC of Vancomycin by BMD to disc diffusion resistant CoNS and *S. aureus* showed that 80% of tested isolates had Vancomycin MIC results of ≤1 µg/ml. Two (13.3%) *S. aureus* and 1 (16.7.4%) CoNS isolate showed MIC results of ≥1.5 µg/ml. Only one (6.7%) *S. aureus* isolates alone showed MIC results of ≥2 µg/ml which is intermediate resistant, where as the rest *S. aureus* and all CoNS and were sensitive to vancomycin as per the 2014 guideline of CLSI.

## Discussion

Bacteria isolation rate in this study 28%, was comparable with the result done in Gonder, Ethiopia, 24.2% (Ali and Kebede 2008), but higher than Addis Ababa, Ethiopia 21.4% (Asrat and Amanuel 2001), Jimma, Ethiopia (8.8%) (Zenebe et al. 2011), Gonder, Ethiopia 18.2% (Dagnew et al. 2013), Nigeria 18.2% (Nwadioha et al. 2010), Malawi 17.6% (Archibald et al. 2000), Nepal 23.1% (Amatya et al. 2007), India 9.94% (Manjula et al. 2005) and Nepal 10% (Usha and Pushpa 2007). It was, however; lower than reports from Zimbabwe 37.1% (Obi and Mazarura 1996), Gambia 34% (Philip et al. 2007) and India 40% (Neuma and Chitnis 1996). Possible explanation for the difference in prevalence of BSI across countries could be due to the difference in blood culture system, the study design, geographical location, nature of patient population, epidemiological difference of the etiological agents, seasonal variations, difference in infection control policies between nations (Gohel et al. 2014; Zenebe et al. 2011; Dagnew et al. 2013).

In our present study, 72.2% of infections were caused by gram-positive and 27.8% by gram-negative bacteria. Similar finding was seen from studies in Gonder, Ethiopia (69 and 31%) (Dagnew et al. 2013), Jimma, Ethiopia, (60.9 and 39.1%) (Zenebe et al. 2011) Gondar, Ethiopia (70.2 and 29.8%) (Ali and Kebede 2008), Zimbabwe (71.9 and 28.1%) (Obi and Mazarura 1996), Addis Ababa, Ethiopia (62.6 and 37.4%) (Shitaye et al. 2010).

On the contrary, gram-negative bacteria have been reported as the commonest cause of bacteraemia among febrile patients in Nigeria (69.3 and 30.7%) (Nwadioha et al. 2010), Saudi Arabia (62.2 and 33.8%) (Elbashier et al. 1998), Tanzania (69.7, 30.3%) (Meremo et al. 2012) and Nepal (89.19 and 10.81%) (David et al. 2004). Possible explanation for the difference could be due to epidemiological difference of the etiological agents.

*S. aureus*, CoNS, *E. coli*, *S. typhi*, *Citrobacter* spp., S. *pyogenes* and *Acinetobacter* spp. were the most common bacterial pathogens causing bacteraemia in this study. More or less similar observations have been seen in cases of bacteraemia in different countries, though, the proportion and prevalence of the bacterial agents varied (Dagnew et al. 2013; Asrat and Amanuel 2001; Zenebe et al. 2011; Ali and Kebede 2008; Obi and Mazarura 1996; Elbashier et al. 1998; Wisplinghoff et al. 2004).

*S. aureus* (37.5%) were the most commonly isolated bacteria in contrary to the other studies (Dagnew et al. 2013; Asrat and Amanuel 2001; Rina et al. 2007; Ali and Kebede 2008; Obi and Mazarura 1996) where coagulase negative staphylococci was the most isolated and in Tanzania, *Salmonella* spp., were reported as the dominant bacteria (Meremo et al. 2012). In our study, the second most bacterial isolate was CoNS (30.6%).This prevalence was comparable to the study done in other parts of Ethiopia 26.1% (Ali and Kebede 2008), 33.3% (Dagnew et al. 2013), but lower than reports from Addis Ababa Ethiopia (43.3%) (Shitaye et al. 2010) and Zimbabwe (42.9%) (Obi and Mazarura 1996). Though CoNS were mainly recognized as a contaminant until the 1970s, studies have reported an increasing incidence of infections due to these bacteria (Dagnew et al. 2013; Boisson et al. 2002).

In this study, *E. coli* (10.4%) was the predominant gram negative bacteria followed by *S. typhi* and *Citrobacter* spp. (5.6% each). However, results from other parts of Ethiopia (Dagnew et al. 2013; Asrat and Amanuel 2001) reported that *Klebsiella* spp. and *E. coli* as the dominant isolates. In contrary to other studies (Asrat and Amanuel 2001; Zenebe et al. 2011; Ali and Kebede 2008; Usha and Pushpa 2007), we have not isolated *H. influenzae* in our study which in lines with the finding from Gonder (Dagnew et al. 2013). In our current study we found all cases of BSI with single microorganism which in lines with earlier reports (Dagnew et al. 2013; Ghanshyam et al. 2008; Angyo et al. 2001). Unlike to our study; however, septicaemia of poly-microbial aetiology were reported by other studies (Obi and Mazarura 1996; Ghanshyam et al. 2002).

In this study bloodstream infection was not age dependent which was in contrast to other previous reports that showed septicaemia was relatively higher in neonates (Dagnew et al. 2013; Komolafe and Adegoke 2008; Shitaye et al. 2010).This may be due to small numbers of children in our study participants.

Overall the resistance of gram positive bacteria was from 0 to 83%, and for gram negative from 0 to 100% which is similar to the result from Jimma which was 0–85.7% and 0–100% for gram negative and positive, respectively (Zenebe et al. 2011). This was however, different from the study result done in Addis Ababa, where the rate for gram positive bacteria ranged from 12 to 76%, and for gram negatives ranged from 8 to 46% (Asrat and Amanuel 2001) and Gonder 23.5–58.8% and 20–100% for gram positive and negative, respectively (Dagnew et al. 2013). The increased resistant blood isolates in this study may be a signal of indiscriminate and continuous use of sub-therapeutic doses of commonly available antimicrobials both in the veterinary and public health sectors (Zenebe et al. 2011). This could challenge the management of patients very difficult.

Amoxicillin clavulanic acid was found to be effective against both gram positive and gram negative isolates in this study. Other studies reported Ciprofloxacin as an effective (Dagnew et al. 2013; Asrat and Amanuel 2001; Zenebe et al. 2011). Ciprofloxacin was found to be effective against gram negative isolates comparable with findings from Ethiopia and other nations (Dagnew et al. 2013; Komolafe and Adegoke 2008; Shitaye et al. 2010).

Antibiograms resistance pattern of the isolates revealed 59% them showed multidrug resistance. This suggests a high resistance gene pool perhaps due to gross misuse and inappropriate usage of the antibacterial agents (Komolafe and Adegoke 2008).

Except one isolate of *S. aureus* which was intermediate resistant, all MICs tested CoNS and *S. aureus* were sensitive to Vancomycin as per the CLSI, 2014 guideline, which was similar to other studies done elsewhere (Sakoulas et al. 2004; Soriano et al. 2008). Possible explanation for the absence of vancomycin resistant bacteria in our study is due to the fact that use of vancomycin by doctors is restricted in the treatment of patients in the study area and in the nation as whole. Unlike to this; however, researchers have reported vancomycin resistant strains from France (Ploy et al. 1998), Korea (Kim et al. 1998), South Africa (Ferraz et al. 2000) and Nigeria (Moses et al. 2013) showing that it is becoming a global threat.

There are also other reports that indicated that there is an increasing vancomycin MICs over time at individual institutions. However, as most clinical laboratories use automated systems to carry out susceptibility testing, these systems do not provide an accurate vancomycin MICs. Hence it has been recommended that clinical laboratories to provide accurate and reliable vancomycin MICs report to clinicians to help select appropriate therapy for the management of patients (Hsu et al. 2008; Steinkraus et al. 2007).

In conclusion, prevalence of BSI in this current study was high. This study added to the knowledge of the epidemiology of the isolates with high rates of resistance to most used antibiotics. Therefore timely investigation of bacterial flora of the bloodstream infections and monitoring of their antibiotic susceptibility pattern is important to reduce the incidence of bloodstream infections and multi drug resistant strains.
